# MicroRNA-27a promotes podocyte injury via PPAR*γ*-mediated *β*-catenin activation in diabetic nephropathy

**DOI:** 10.1038/cddis.2017.74

**Published:** 2017-03-09

**Authors:** Zhanmei Zhou, Jiao Wan, Xiaoyan Hou, Jian Geng, Xiao Li, Xiaoyan Bai

**Affiliations:** 1 Division of Nephrology, Nanfang Hospital, Southern Medical University, National Clinical Research Center for Kidney Disease, State Key Laboratory of Organ Failure Research, Guangdong Provincial Institute of Nephrology, Guangzhou, Guangdong, People's Republic of China; 2 Department of Nephrology, The First Affiliated Hospital, Inner Mongolia Medical University, Hohhot, Inner Mongolia, People's Republic of China; 3 Department of Pathology, Nanfang Hospital, Southern Medical University, Guangzhou, Guangdong, People's Republic of China; 4 Department of Emergency, Nanfang Hospital, Southern Medical University, Guangzhou, Guangdong, People's Republic of China

## Abstract

Podocyte injury has a pivotal role in the pathogenesis of diabetic nephropathy (DN). MicroRNA-27a (miR-27a), peroxisome proliferator-activated receptor *γ* (PPAR*γ*) and *β*-catenin pathways have been involved in the pathogenesis of DN. Herein, we asked whether miR-27a mediates podocyte injury through PPAR*γ/β*-catenin signaling in DN. The functional relevance of miR-27a, PPAR*γ* and *β*-catenin were investigated in cultured podocytes and glomeruli of diabetic rats and patients using *in vitro* and *in vivo* approaches. Podocyte injury was assessed by migration, invasion and apoptosis assay. Biological parameters were analyzed using enzyme-linked immunosorbent assay. We found that high glucose stimulated miR-27a expression, which, by negatively targeting PPAR*γ*, activated *β*-catenin signaling as evidenced by upregulation of *β*-catenin target genes, snail1 and *α*-smooth muscle actin (*α*-SMA) and downregulation of podocyte-specific markers podocin and synaptopodin. These changes caused podocyte injury as demonstrated by increased podocyte mesenchymal transition, disrupted podocyte architectural integrity and increased podocyte apoptosis. Furthermore, we provide evidence that miR-27a contributed to unfavorable renal function and increased podocyte injury in diabetic rats. Notably, miR-27a exhibited clinical and biological relevance as it was linked to elevated serum creatinine, proteinuria and reduced creatinine clearance rate. In addition, miR-27a upregulation and activation of PPAR*γ/β*-catenin signaling were verified in renal biopsy samples from DN patients. We propose a novel role of the miR-27a/PPAR*γ/β*-catenin axis in fostering the progression toward more deteriorated podocyte injury in DN. Targeting miR-27a could be a potential therapeutic approach for DN.

Diabetes mellitus, one of the fastest growing metabolic disorders in the world, contributes to about 3.2 million diabetes-related deaths annually.^1, 2^ Diabetic nephropathy (DN) is the most common cause of end-stage renal disease and cardiovascular events.^3, 4^ In the past decades, although the first-line therapy such as angiotensin-converting enzyme inhibitors/angiotensin II receptor blockers can slow but cannot stop the progression of DN, which urgently calls for innovative therapeutic strategies.^5, 6^

Adult podocytes are unique terminally differentiated glomerular epithelial cells critical in maintaining the integrity of the glomerular filtration barrier.^7^ Reduced podocyte number leads to proteinuria and glomerulosclerosis in diabetic^8^ and non-diabetic glomerular diseases.^9^ Loss of podocyte-specific molecules, such as podocin, synaptopodin and nephrin, causes podocyte injury and epithelial to mesenchymal transition.^10, 11^

Accumulating evidence has demonstrated that *β*-catenin activation is closely related to the emergence and development of various disease pathologies.^12, 13^ The pathway activates downstream target genes, such as snail1, c-myc and *α*-smooth muscle actin (*α*-SMA), thereby regulating many biological processes through a complex of *β*-catenin and the T-cell factor/lymphoid-enhancer factor 1 (TCF/LEF1) family.^14^ Wnt stabilizes cytosolic *β*-catenin, which then binds to TCF/LEF1 in the nucleus and recruits transcription factors Brg1 and CREB-binding protein to initiate Wnt-targeted gene expression.^15, 16^ *β*-Catenin activation triggers the activities of downstream transcription factors and activates the mesenchymal transition of podocytes.^17, 18^

Peroxisome proliferator-activated receptor gamma (PPAR*γ*) is an important regulator of cell proliferation and differentiation.^19^ Our previous study has revealed that high glucose stimulated PPAR*γ* phosphorylation in cultured renal tubular epithelial cells.^20^ Other studies have shown that activation of PPAR*γ* can promote the degradation of *β*-catenin in mesenchymal stem cells and direct interaction with *β*-catenin.^21^ Recently, a PPAR*γ* region and *β*-catenin binding domain were identified to have high homology with TCF/LEF, which facilitates its interaction with *β*-catenin.^22^ Undoubtedly, the PPAR*γ/β*-catenin signaling is drawing increasing attention and has a critical role in the pathogenesis of diseases.

MicroRNAs (miRNAs) regulate gene expression after recognition of specific binding to the 3′-untranslated region (UTR) of target mRNAs, causing mRNA deadenylation and degradation or translational repression.^23^ Alterations of gene expression resulting from aberrant expression of miRNAs are associated with numerous diseases.^24, 25^

Along with others, we have shown that microRNA-27a (miR-27a), by targeting PPAR*γ*, exacerbates renal tubulointerstitial fibrosis and mesangial cell injury in DN.^20, 26^ In this study, our aim was to explore whether miR-27a promotes podocyte injury through PPAR*γ*-mediated *β*-catenin activation in DN.

## Results

### High glucose induces miR-27a expression in cultured podocytes

**Figure 1:**
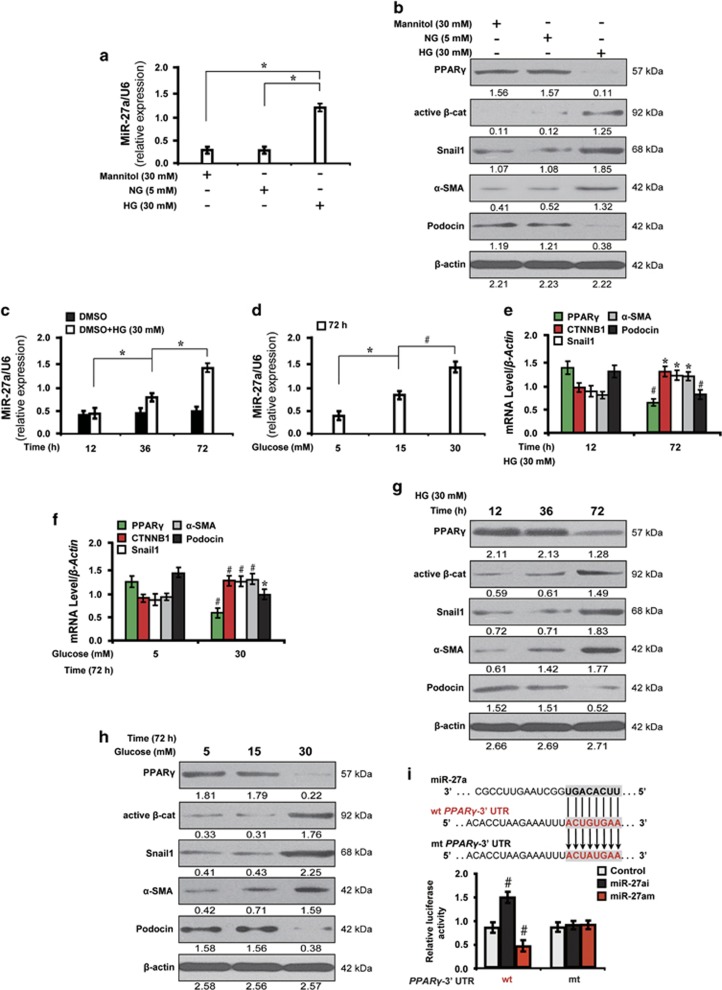
High glucose induces miR-27a expression in cultured podocytes. (**a**) qRT-PCR analysis shows the level of miR-27a in various conditions as indicated. (**b**) Representative western blotting shows the expression of PPAR*γ* and *β*-catenin target genes in various conditions as indicated. Cell lysates were immunoblotted with specific antibodies against PPAR*γ*, active *β*-catenin, snail1, *α*-SMA, podocin and *β*-actin. qRT-PCR shows that miR-27a was increased in a (**c**) time and (**d**) dose-dependent manner. (**e-f**) qRT-PCR and (**g-h**) western blot analyses show the expression level of PPAR*γ* and *β*-catenin target genes in a time- and dose-dependent manner. (**i**) PPAR*γ* gene transcription was amplified by miR-27ai and diminished by miR-27am. Mouse podocytes were co-transfected with miR-27ai, miR-27am, and control or wild-type or mutant 3′-UTR of PPAR*γ* and transfection efficiency was evaluated by luciferase reporter assay. *P<0.05; ^#^*P*<0.001. Active *β*-cat, active *β*-catenin; CTNNB1, catenin beta-1; HG, high glucose; miR-iNC: miRNA inhibitor negative control; mt: mutant type; NG, normal glucose; wt: wild type

To investigate the effect of high glucose stimulation on the expression of miR-27a and downstream genes, podocytes were cultured in different conditions and biological behaviors were observed. First, the level of miR-27a was increased in high glucose (30 mM) cultured podocytes as determined by quantitative real-time RT-PCR (qRT-PCR) analysis (Figure 1a). As shown in Figure 1b, high glucose suppressed the expression of PPAR*γ* and podocyte-specific marker podocin, but activated *β*-catenin and its target genes, snail1 and *α*-SMA. Mannitol (30 mM), however, had no effect on the expression of these molecules. Next, we investigated the effect of time and glucose concentration on miR-27a expression. We found that miR-27a was increased in a time-dependent (Figure 1c) and dose-dependent (Figure 1d) manner as detected by qRT-PCR. Then, we examined whether miR-27a modulated PPAR*γ*-mediated downstream gene expression levels in high glucose. As illustrated by qRT-PCR (Figures 1e and f) and western blot analyses (Figures 1g and h), the decreased expression level of PPAR*γ* was time and dose dependent. In addition, *β*-catenin and its target genes, snail1 and *α*-SMA, were markedly increased and podocyte-specific marker podocin was downregulated. We finally asked whether miR-27a-induced downstream changes depended on PPAR*γ*. Of note, the luciferase activity of wild-type 3′-UTR of PPAR*γ* was significantly increased when treated with miR-27a inhibitor (miR-27ai) but decreased with miR-27a mimics (miR-27am) as compared with the mutant (Figure 1i), indicating that PPAR*γ* is a direct downstream target of miR-27a. These results indicate a correlation between miR-27a and PPAR*γ*-mediated downstream gene expression levels in high glucose cultured podocytes.

### MiR-27a promotes podocyte injury via PPAR*γ*-mediated *β*-catenin activation in high glucose

**Figure 2:**
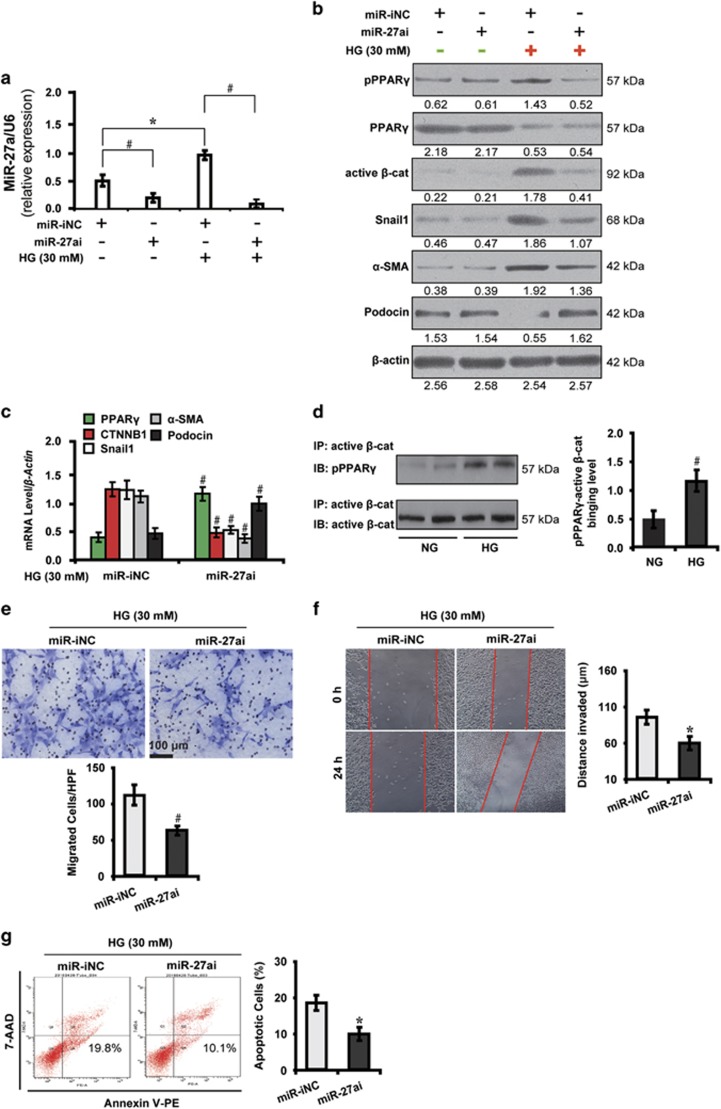
MiR-27ai attenuates podocyte injury via PPAR*γ*-mediated *β*-catenin inactivation in high glucose. (**a**) qRT-PCR analysis shows miR-27ai reduced miR-27a expression in HG cultured podocytes. (**b**) Representative western blotting shows the expression of phosphorylated and total PPAR*γ* and *β*-catenin target genes in various conditions as indicated. (**c**) qRT-PCR analysis shows miR-27ai upregulated the level of PPAR*γ* and podocin but downregulated *β*-catenin target genes. (**d**) HG enhanced the interaction of phosphorylated PPAR*γ* and active *β*-catenin by co-immunoprecipitation. (**e**) Transwell migration assay and quantitative data show decreased migration of HG cultured podocytes. Scale bar, 100 *μ*m. (**f**) Wound-healing assay and quantitative data show decreased invasion of HG cultured podocytes. (**g**) Flow cytometric analysis shows decreased podocyte apoptosis. *P<0.05; ^#^*P*<0.001. Active *β*-cat, active *β*-catenin; 7-AAD, 7-aminoactinomycin D; CTNNB1, catenin beta-1; HG, high glucose; IB, immunoblotting; IP, immunoprecipitation; miR-iNC: miRNA inhibitor negative control; miR-NC: miRNA negative control; NG, normal glucose; PE, phycoerythrin; pPPAR*γ*, phosphorylated peroxisome proliferator-activated receptor *γ*

Having discovered the upregulation of miR-27a in high glucose condition, we next explored the underlying mechanisms of miR-27a-induced podocyte injury. As shown in Figure 2a, miR-27ai decreased miR-27a expression in podocytes by qRT-PCR analysis. Furthermore, high glucose-stimulated PPAR*γ* phosphorylation was attenuated by miR-27ai (Figure 2b). In addition, miR-27ai inactivated the expression of *β*-catenin and its target genes, snail1 and *α*-SMA, but increased podocin level in high glucose instead of the normal glucose condition as shown by western blot (Figure 2b) and qRT-PCR analyses (Figure 2c). These data suggest underlying mechanisms may be involved in the post-translational modification of PPAR*γ*.

This observation led us to ask whether PPAR*γ* physically interacted with *β*-catenin in mediating the downstream events. To test this hypothesis, we carried out co-immunoprecipitation analysis and examined the interaction between phosphorylated PPAR*γ* and active *β*-catenin. As shown in Figure 2d, physical interaction between phosphorylated PPAR*γ* and active *β*-catenin were detected in podocytes and was strengthened by high glucose stimulation. Strikingly, miR-27ai rendered podocytes less vulnerable to the deleterious effects of high glucose concentrations, leading to decreased abilities of migration (Figure 2e), invasion (Figure 2f) and decreased apoptosis (Figure 2g). In contrast, miR-27a enrichment with miR-27am had the opposite effects (Supplementary Figures 1a–f). These data collectively demonstrate that miR-27a, via activation of PPAR*γ/β*-catenin signaling, induces high glucose cultured podocytes to undergo mesenchymal transition and apoptosis, hence incurring podocyte injury.

### PPAR*γ*-mediated *β*-catenin activation triggers podocyte injury in high glucose

**Figure 3:**
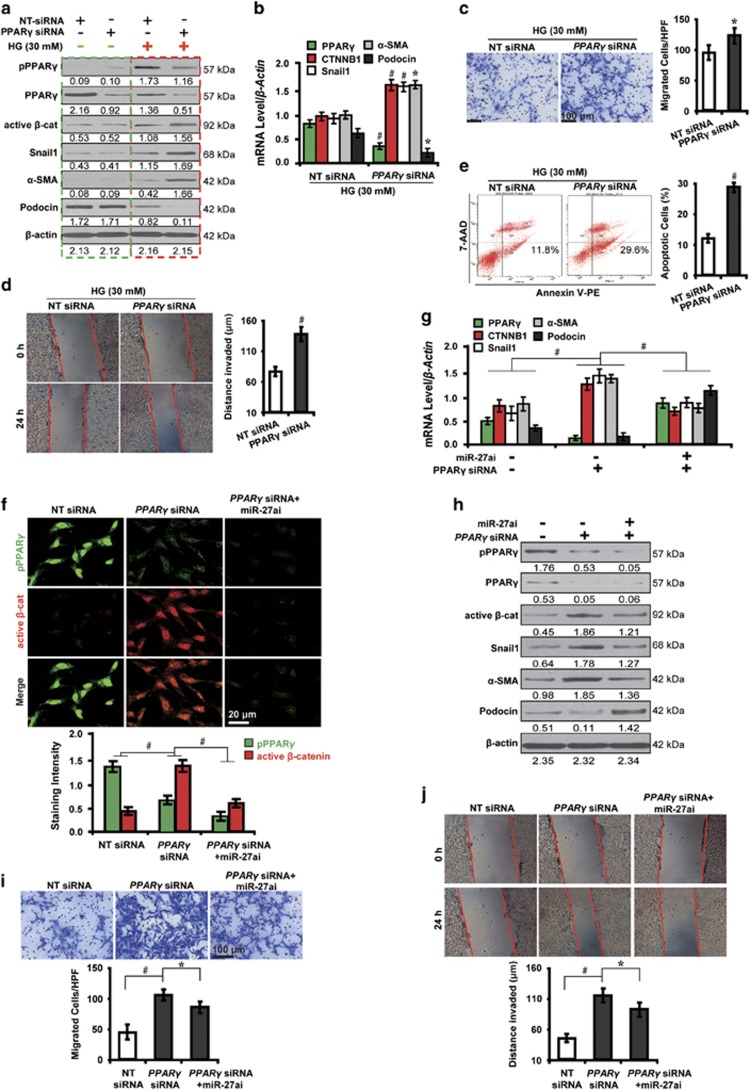
PPAR*γ*-mediated *β*-catenin activation induces podocyte injury in high glucose. (**a**) Representative western blotting shows the expression of phosphorylated and total PPAR*γ* and *β*-catenin target genes in various conditions as indicated. (**b**) qRT-PCR analysis shows PPAR*γ* siRNA decreased PPAR*γ* and podocin but increased *β*-catenin target genes. (**c**) Transwell migration assay and quantitative data show increased migration. Scale bar, 100 *μ*m. (**d**) Wound-healing assay and quantitative data show increased invasion. (**e**) Summarized data showing increased podocyte apoptosis by flow cytometric analysis. (**f**) Immunofluorescence microscopy and quantitative data show PPAR*γ* siRNA-induced *β*-catenin activation was attenuated by co-transfection with miR-27ai. Scale bar, 20 *μ*m. Representative (**g**) qRT-PCR and (**h**) western blotting show the expression of PPAR*γ* and *β*-catenin target genes in various conditions as indicated. Representative (**i**) transwell migration assay and (**j**) wound-healing assay show the increased ability of migration and invasion caused by PPAR*γ* abolishment were mitigated by co-transfection with miR-27ai. *P<0.05; ^#^*P*<0.001. Active *β*-cat, active *β*-catenin; CTNNB1, catenin beta-1; NT, non-targeting; pPPAR*γ*, phosphorylated peroxisome proliferator-activated receptor *γ*

To decipher whether PPAR*γ* mediates Wnt/*β*-catenin signaling and podocyte injury in high glucose, we further examined the function of PPAR*γ* using loss- and gain-of-function studies with PPAR*γ* siRNA or its agonist rosiglitazone.^27^ Notably, we found that in high glucose condition, PPAR*γ* siRNA decreased PPAR*γ* phosphorylation, activated *β*-catenin and its target genes, snail1 and *α*-SMA, and downregulated podocin (red dashed box, Figure 3a). These changes, however, were not detected in normal glucose (green dashed box, Figure 3a). As shown by qRT-PCR analysis (Figure 3b), in high glucose, PPAR*γ* depletion decreased the mRNA level of PPAR*γ* and podocin, and increased *β*-catenin and its target genes, snail1 and *α*-SMA. These results suggest that a high glucose microenvironment is essential for PPAR*γ* phosphorylation. Furthermore, PPAR*γ* knockdown promoted the migration and invasion ability of high glucose cultured podocytes (Figures 3c and d) and increased apoptosis (Figure 3e). Conversely, PPAR*γ* overexpression with rosiglitazone exerted the opposite effects (Supplementary Figures 2a–e). Taken together, these results indicate the pivotal role of PPAR*γ* in mediating podocyte injury via activating Wnt/beta-catenin signaling in high glucose.

Having shown a reciprocal interplay between miR-27a and PPAR*γ*, we next investigated whether the effect of miR-27a on downstream gene expression levels and podocyte functions depended on *PPARγ*. To this end, high glucose cultured podocytes were co-transfected with *PPARγ* siRNA and miR-27ai. As shown by immunofluorescence microscopy (Figure 3f), *PPARγ* siRNA-induced *β*-catenin activation, which was attenuated by co-transfection with miR-27ai. In this context, we hypothesized that PPAR*γ* indispensably mediated *β*-catenin-induced downstream gene expression levels. This hypothesis was verified as exemplified by downregulation of *β*-catenin target genes, snail1 and *α*-SMA, and podocin upregulation by qRT-PCR (Figure 3g) and western blot analyses (Figure 3h). In addition, the increased ability of migration (Figure 3i) and invasion (Figure 3j) induced by PPAR*γ* abolishment were mitigated by co-transfection with miR-27ai. Collectively, these results suggest that *PPARγ* is required in miR-27a-induced *β*-catenin activation and podocyte injury in high glucose.

### MiR-27a contributes to worsened renal function and PPAR*γ*-mediated *β*-catenin activation in diabetic rats

**Figure 4:**
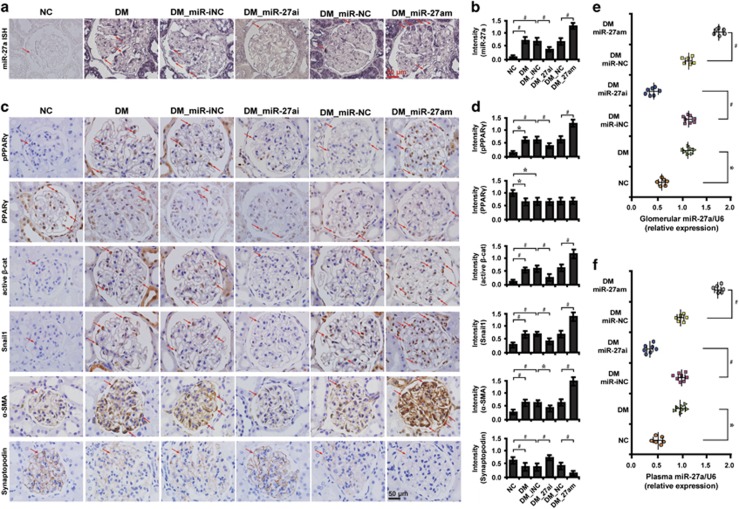
MiR-27a promotes PPAR*γ*-mediated *β*-catenin activation in diabetic rats. Representative (**a**) micrographs of ISH and (**b**) quantitative data illustrate the expression of miR-27a in podocytes in various groups as indicated. Representative (**c**) immunohistochemical staining and (**d**) quantitative data show the expression of PPAR*γ* and *β*-catenin target genes in various groups as indicated. The level of miR-27a in (**e**) glomeruli and (**f**) plasma samples of diabetic rats in various groups as indicated. Frozen rat kidney sections were hybridized with digoxygenin (DIG)-labeled miRCURY Locked Nucleic Acid (LNA) microRNA Detection Probes. Paraffin-embedded sections were immunostained for PPAR*γ* (total and phosphorylated), active *β*-catenin, snail1, *α*-SMA and synaptopodin. Scale bar, 50 *μ*m. Glomeruli were dissected using laser microdissection and detected with qRT-PCR. *P<0.05; ^#^*P*<0.001. Active *β*-cat, active *β*-catenin; DM, diabetes mellitus; DM_miR-iNC, diabetic rats treated with miRNA inhibitor negative control; DM_miR-27ai, diabetic rats treated with miR-27ai; NC, normal control; pPPAR*γ*, phosphorylated peroxisome proliferator-activated receptor *γ*

We finally investigated the *in vivo* relevance of the interplay between miR-27a and PPAR*γ/β*-catenin signaling. MiR-27ai or miR-27am were administered to diabetic rats and the functional effects examined. We found that miR-27ai significantly improved renal function as evidenced by decreased level of serum creatinine (Scr), serum blood urea nitrogen (BUN), urine albumin excretion rate (UAER), urine albumin to creatinine ratio (UACR) and increased creatinine clearance rate (Ccr) (Table 1). Conversely, miR-27am exerted opposite effects (Table 2). To confirm the localization of miR-27a in glomeruli, we next examined miR-27a expression using *in situ* hybridization (ISH). Compared with normal control rats, miR-27a was upregulated in podocytes of diabetic rat kidney tissues, as well as in renal tubular epithelial cells, which was significantly abolished by miR-27ai and enriched by miR-27am (Figures 4a and b). Furthermore, miR-27ai reduced PPAR*γ* phosphorylation and *β*-catenin activation in podocytes. Concomitantly, the expression of *β*-catenin target genes snail1 and *α*-SMA were downregulated and podocyte-specific marker synaptopodin was markedly upregulated. MiR-27am, however, had the opposite effects (Figures 4c and d). Further, elevated miR-27a level was found in microdissected glomeruli (Figure 4e) and plasma samples (Figure 4f) of diabetic rats, which was decreased by treatment with miR-27ai and increased by miR-27am. These data further illustrate the functional interplay between miR-27a and PPAR*γ/β*-catenin signaling in diabetic rats *in vivo*.

### MiR-27a causes podocyte depletion and disrupts its architectural integrity in diabetic rats

**Figure 5:**
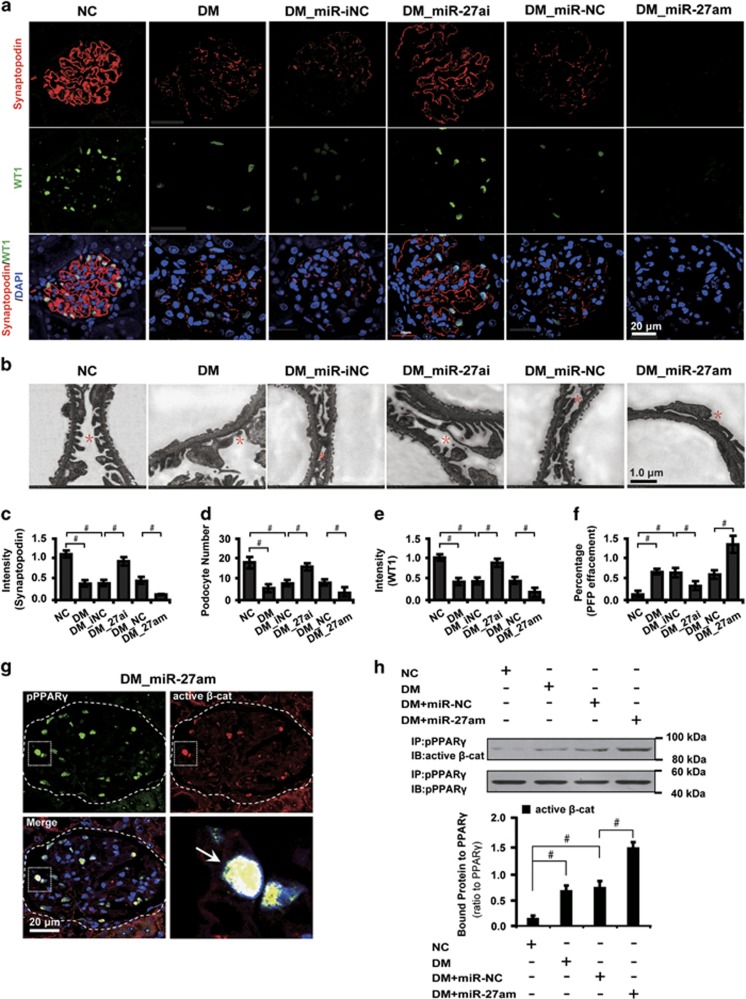
miR-27a contributes to podocyte depletion and disrupts podocyte architectural integrity in diabetic rats. (**a**) Immunofluorescence staining shows glomerular synaptopodin and WT1 expression in different groups. Paraffin-embedded rat kidney sections were co-immunostained for synaptopodin (red) and WT1 (green). Nuclei were visualized by DAPI. Scale bar, 20 *μ*m. (**b**) Transmission electron microscopic analysis shows morphological changes in the podocyte foot process in different groups as indicated. Scale bar, 1.0 *μ*m. Red asterisks specify podocyte foot processes. (**c**) Level of synaptopodin, (**d**) podocyte number, (**e**) level of WT1 and (**f**) percentage of podocyte foot process effacement in different groups were shown. (**g**) Representative immunofluorescence micrographs show glomerular pPPAR*γ* and active *β*-catenin expression in diabetic rats with magnified white insets on the lower right quadrant. Paraffin-embedded rat kidney sections were co-immunostained for pPPAR*γ* (green) and active *β*-catenin (red). Nuclei were visualized by DAPI. Arrows indicate the colocalization (yellow). Scale bar, 20 *μ*m. (**h**) Representative co-immunoprecipitation analysis shows the interaction of pPPAR*γ* and active *β*-catenin in laser capture microdissected glomeruli in different groups as indicated. *P<0.05; ^#^*P*<0.001. Active *β*-cat, active *β*-catenin; DM, diabetes mellitus; DM_miR-iNC, diabetic rats treated with miRNA inhibitor negative control; DM_miR-27ai, diabetic rats treated with miR-27ai; IB, immunoblotting; IP, immunoprecipitation; NC, normal control; pPPAR*γ*, phosphorylated peroxisome proliferator-activated receptor *γ*; WT1, Wilm's tumor 1

Given that miR-27a induces abolishment of slit diaphragm-associated protein synaptopodin, we next examined whether miR-27a affected podocyte number and architectural integrity. We found that in diabetic glomeruli, podocyte architectural integrity was disrupted as evidenced by a decrease of the podocyte marker synaptopodin, loss of WT1-positive podocytes and effaced podocyte foot processes. These changes were mitigated by miR-27ai and exaggerated by miR-27am (Figures 5a–f). Next, we asked whether miR-27a-dictated podocyte injury depended upon the interplay between PPAR*γ* and *β*-catenin activation. To this end, we carried out immunofluorescence microscopy and co-immunoprecipitation analyses to further explore their correlations. The results showed that phosphorylated PPAR*γ* and active *β*-catenin colocalized and interacted with each other (Figure 5g), which was strengthened both in diabetic conditions and by miR-27am treatment (Figure 5h). These results together suggest that miR-27a induces podocyte injury via the PPAR*γ/β*-catenin signaling.

### Expression patterns of miR-27a and PPAR*γ/β*-catenin-related markers in renal biopsies from DN patients

**Figure 6:**
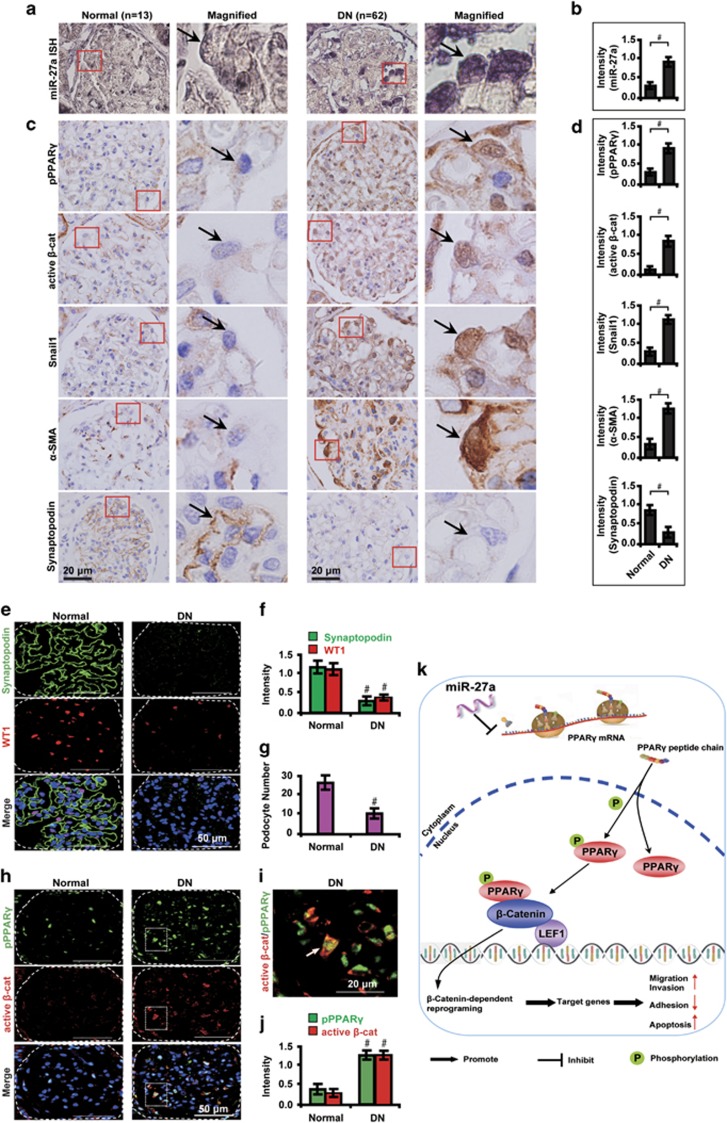
Expression patterns of miR-27a and PPAR*γ/β*-catenin signaling molecules in human renal biopsy samples. Representative (**a**) ISH staining and (**b**) quantitative data illustrate the expression of miR-27a in human renal biopsy tissues from DN patients (*n*=62) and control healthy donor transplant kidney tissues (*n*=13). Representative (**c**) immunohistochemical staining and (**d**) quantitative data illustrate the expression of pPPAR*γ* and *β*-catenin target genes in different groups as indicated. Fresh snap-frozen human renal biopsies were sectioned and hybridized with digoxygenin (DIG)-labeled miRCURY Locked Nucleic Acid (LNA) microRNA Detection Probes. Paraffin-embedded human renal biopsy sections were immunostained for pPPAR*γ*, active *β*-catenin, snail1, *α*-SMA and synaptopodin. Red insets in the left side images of each panel were magnified and shown on the right side. Black arrows indicate podocytes. Representative (**e**) immunofluorescence images and quantitative data show (**f**) glomerular synaptopodin (green) and WT1 (red) expression and (**g**) podocyte number in human renal biopsy samples. (**h**) Representative immunofluorescence micrographs show glomerular pPPAR*γ* (green) and active *β*-catenin (red) expression in human renal biopsy samples. Scale bar, 50 *μ*m. (**i**) Magnified white insets show the colocalization of pPPAR*γ* and active *β*-catenin (yellow). Scale bar, 20 *μ*m. (**j**) Quantitative data show the level of pPPAR*γ* (green) and active *β*-catenin (red). Nuclei were visualized by DAPI. White arrow indicates colocalization of pPPAR*γ* and active *β*-catenin. Glomeruli were demarcated with white dashed lines. (**k**) A hypothetical model illustrating that miR-27a, via PPAR*γ*-mediated *β*-catenin activation, promotes podocyte injury in DN. MiR-27a inhibits PPAR*γ* gene transcription whereas indirectly stimulates PPAR*γ* phosphorylation, which activates *β*-catenin signaling and triggers *β*-catenin-dependent reprogramming and target gene expression levels. These events promote podocyte injuries as demonstrated by increased migration, invasion, and apoptosis, and decreased adhesion abilities. #*P*<0.001. Active *β*-cat, active *β*-catenin; LEF1, lymphoid-enhancer factor 1; P, phosphorylation; pPPAR*γ*, phosphorylated peroxisome proliferator-activated receptor *γ*; WT1, Wilm's tumor 1

We verified the expression patterns of miR-27a and PPAR*γ/β*-catenin-related markers in human renal biopsy samples from DN patients. Similar to changes detected in animal studies, compared with healthy transplant donor kidney biopsies, ISH analysis shows that miR-27a level was upregulated in podocytes of renal biopsies from DN patients (Figures 6a and b). Concomitantly, activation of phosphorylated PPAR*γ* and *β*-catenin were observed in podocytes of renal tissues from DN patients. Consistently, *β*-catenin activation also led to increased expression of snail1 and *α*-SMA but decreased synaptopodin (Figures 6c and d). In addition, podocyte injury was demonstrated by loss of the slit diaphragm-associated protein synaptopodin and decreased number of WT1-positive podocytes as detected by immunofluorescence microscopy (Figures 6e–g). Colocalization of phosphorylated PPAR*γ* and active *β*-catenin was also identified in podocytes of DN samples (Figures 6h–j), further supporting the notion that PPAR*γ*-mediated *β*-catenin activation promotes podocyte injury in DN. A hypothetical model illustrated that miR-27a, via PPAR*γ*-mediated *β*-catenin activation, promotes podocyte injury in DN (Figure 6k).

## Discussion

Podocytes, the terminally differentiated visceral epithelial cells, uniquely reside along the glomerular basement membrane.^28, 29^ Podocyte foot processes form one of the most critical components of glomerular filtration barrier and keep the integrity of the glomerulus.^30^ Pathologically, multiple injurious stimuli have been found to contribute to podocyte dedifferentiation, featured by loss of podocyte-specific properties and gain of mesenchymal features, thus leading to podocyte dysfunction and defects of glomerular filtration barrier.^31^ Diabetes is a condition in which podocytes are universally exposed to high glucose and its resulting metabolites, such as advanced glycation end products (AGEs) and oxidative stress.^23, 32, 33^

Several miRNAs, such as microRNA-130b,^34^ microRNA-182,^35^ microRNA-146a^36^ and microRNA-200a,^37^ have been found dysregulated in diabetes. They function as a ubiquitous class of noncoding RNA modulators of mammalian physiological responses acting via posttranscriptional regulation of gene expression.^38^ In this study, we have identified that high glucose induces the expression of miR-27a in cultured podocytes. In addition, miR-27a upregulation has also been detected in animal models of diabetes and DN patients. This finding highlights the significance of miR-27a in podocyte pathophysiology in DN.

The most novel finding in this study is that in DN miR-27a induces podocyte injuries and worsens renal function via PPAR*γ*-mediated *β*-catenin activation. This conclusion is authenticated by several lines of evidence. First, miR-27a directly targets the 3′-UTR of *PPARγ* mRNA and provokes the PPAR*γ*-mediated downstream events (Figure 1). Second, PPAR*γ* phosphorylation causes *β*-catenin activation, which triggers a series of *β*-catenin-dependent reprogramming in podocytes, such as enhanced epithelial–mysenchymal transition, loss of podocyte-specific markers and increased apoptosis (Figures 2 and 3). Third, miR-27a disrupts podocyte architectural integrity and reduces podocyte number in diabetic rats. Finally, miR-27a deteriorates renal function as revealed by elevated albuminuria and dropped Ccr in diabetic rats. The finding that miR-27a targets PPAR*γ* and controls the expression of *β*-catenin target genes provides novel and mechanistic insights into how miRNAs contribute to the development and progression of DN. This notion inspires us to develop new combinational therapeutic strategies to simultaneously regulate miR-27a downstream signaling, such as PPAR*γ* and *β*-catenin.

Another intriguing finding in this study is that miR-27a-induced *β*-catenin activation depends on PPAR*γ* phosphorylation in DN. We also found that miR-27ai increased PPAR*γ* transcriptional activity but PPAR*γ* protein level remained unchanged in high glucose. This observation led us to reason it is likely that inhibiting PPAR*γ* phosphorylation is an alternative mechanism for anti-diabetic effects of PPAR*γ* ligands. Indeed, this speculation has been supported by emerging lines of evidence showing that many PPAR*γ*-based novel drugs, such as SR1664^39^ and UHC1,^40^ have a separate biochemical activity, blocking the obesity-linked phosphorylation of PPAR*γ* by Cdk5.^20^ These novel synthetic compounds have a unique mode of binding to PPAR*γ* while lacking classical transcriptional agonism, and hence with fewer side effects such as fluid retention, bone fracture, weight gain or congestive heart failure.^41^ We believe that miR-27ai has promising efficacies in inhibiting PPAR*γ* phosphorylation similar to the above mentioned new synthetic compounds. More broadly, the potential of ‘partial agonists' to modulate protein phosphorylation may be feasible, possibly allowing for identification of novel miR-27a targeted drugs. Exactly how miR-27a affects PPAR*γ* phosphorylation is unknown at this stage and warrants additional investigation.

The therapeutic efficacy of miR-27a blockage by its inhibitors results in reversal of the mesenchymal transition and architectural defects of the podocyte. It also combats proteinuria and renal injury in diabetic rats (Figure 4,Tables 1 and 2). This finding is in line with the critical role of *β*-catenin inactivation in a series of studies.^42, 43^ It is also consistent with our previous study illustrating the inhibitory effect of miR-27a/PPAR*γ* axis in renal tubulointerstitial fibrosis in DN.^44^ In glomerular mesangial cells, miR-27a has been reported to induce progression of DN by targeting PPAR*γ*.^26^ The similarities and differences between our present study and the above studies are as follows: (1) by targeting PPAR*γ*, miR-27a could initiate each individual event provoking different downstream signalings in different cell types in diabetic kidney, which may have amplifying effects in exacerbating the disease; (2) miR-27a could contribute to the formation of the most typical pathological hallmark of diabetic glomerular lesions, for example, extracellular matrix accumulation and podocyte foot process effacement; and (3) miR-27a is a robust biomarker in DN, which may be used for evaluating the disease severity. It should also be noted that PPAR*γ* and *β*-catenin are widely expressed in the kidney, so the effects of systemic administration of miR-27ai or miR-27am may affect multiple cell types. Therefore, it raises the topic of combinational therapy in DN in that by synergistically targeting miR-27a/PPAR*γ/β*-catenin and miR-27a/PPAR*γ*/TGF-*β* signalings, both podocyte injuries and tubulointerstitial fibrosis could be reversed or even prevented. It is also possible that by targeting miR-27a alone, *β*-catenin and TGF-*β*^45, 46, 47, 48^ downstream events may be inhibited. Future studies will be needed to explore the efficacies of miR-27ai in clinical settings.

Our study is limited in that we did not tailor the treatment strategy according to the disease stage in the animal studies. Future studies will be needed to explore how to select the appropriate time point for using single/combinational therapies, for example, miR-27ai at the early stage and inhibitors of *β*-catenin and TGF-*β* in later stages. In addition, whether cross-talks between glomerular and tubular cells or cross-talks between podocytes and mesangial cells initiate miR-27a-mediated downstream signalings remains unknown at these stage. These areas warrant further additional exploration.

The results in this study, for the first time, demonstrate that miR-27a/PPAR*γ* axis, as an upstream regulatory signaling, dictates the expression of genes associated with podocyte biology via *β*-catenin pathway. Thus, miR-27a/PPAR*γ* and *β*-catenin are intimately connected to constitute a pathologic axis having a crucial role in the pathogenesis of DN.

In summary, we have shown that miR-27a, via activating PPAR*γ/β*-catenin signaling, exacerbates podocyte injury as evidenced by increased capacity of migration, invasion and apoptosis. In addition, we have identified that miR-27ai is effective in reversing proteinuria and renal dysfunction in DN. This study provides a mechanistic interplay between miR-27a and PPAR*γ/β*-catenin activation in the pathogenesis of DN.

## Materials and Methods

### Cell culture

A conditionally immortalized murine podocyte cell line was maintained as described previously.^49^ Briefly, cells were cultured at 33 °C in RPMI medium 1640 (Sigma-Aldrich, , St. Louis., MO, USA) supplemented with 10% fetal bovine serum (FBS; Life Technologies, Carlsbad, CA, USA) and 10 units/ml mouse recombinant interferon-*γ* (IFN-*γ*, R&D Systems, Minneapolis, MN, USA). To induce differentiation, podocytes were grown under non-permissive conditions at 37 °C in the absence of IFN-*γ* for 14 days before experiments.

Podocytes were then maintained in normal glucose (5 mM) for 1 week, grown to 75–85% confluence and made quiescent by incubation overnight in a serum-free medium. Podocytes were next exposed to mannitol (30 mM) or high glucose (30 mM) for time periods as individual experiments required.

### Luciferase reporter assay

The predicted 3′-UTRs sequence of PPAR*γ* interacting with miR-27a and mutated sequences within the predicted target sites were synthesized and inserted into the pRL-TK control vector (Promega, Madison, WI, USA). Podocytes transfected with 120 ng miR-27ai or negative controls (NCs), followed by co-transfection with 30 ng of the wild-type or mutant 3′-UTR of PPAR*γ* using 0.45 *μ*l of Fugene (Promega). Luciferase assay was carried out on extracts from the cells 48 h post transfection and measured using Dual-Luciferase Assay System (Promega). pRL-TK expressing Renilla luciferase was co-transfected as an internal control. Data were normalized by the ratio of Firefly and Renilla luciferase activities.

### Transfection of miRNA mimics, inhibitors and siRNA

MiR-27ai, miR-27am or the appropriate NCs of miRNA inhibitor (miR-iNC) and miRNA mimics (miR-NC), respectively, were purchased from GenePharma (Shanghai, China) and transfected at a final concentration of 50–100 nM in the cells using HiPerFect Transfection Reagent (Qiagen, Hilden, Germany) according to the manufacturer's recommendations. Expression of murine PPAR*γ* was knocked down with small interfering RNA (siRNA) duplexes using Oligofectamine (Invitrogen, Carlsbad, CA, USA). The target sequence for PPAR*γ* mRNA was: 5′-AAUAUGACCUGAAGCUCCAAGAAUAAG-3′. Non-targeting siRNA pool (D-001206-13-05; Dharmacon, Fisher Scientific, Pittsburgh, PA, USA) was used as a NC. Podocytes were transfected with 1 *μ*g of siRNA in reduced serum medium (OPTI-MEM-I; Invitrogen, Carlsbad, CA, USA) according to the manufacturer's protocol and harvested 72 h post transfection. The RNA and protein were extracted and analyzed, respectively.

### Drug treatment

PPAR*γ* agonist rosiglitazone (Rosi.; 50 nM; R2408; Sigma-Aldrich) was used to treat podocytes. Cells in each group were treated for 72 h and then harvested for further analyses.

### Transwell migration assay

Cells (1.0 × 10^6^ cells/ml) in serum-free medium were added to the top chamber of 24-well transwell plates (8 mm pore size; Corning Star, Cambridge, MA, USA) and 600 *μ*l of complete medium with 10% FBS was in the bottom chamber. The assembled chamber was incubated at 37 °C in a humidified, 5% CO_2_ cell culture incubator for 24 h and fixed with 10% formalin and stained with crystal violet for visualization.

### Wound-healing assay

Podocytes (2.0 × 10^5^ cells per well) were plated in six-well plates and grown to 80% confluence. The individual wells were wounded by scratching with a pipette tip and incubated with serum-free medium to various time points. Wells were photographed under phase-contrast microscopy ( × 10).

### *In vivo* animal studies

All animal experiments were specifically approved by the Animal Ethics Committee at Nanfang Hospital, Southern Medical University, Guangzhou, China. The study protocols conform to the Guide for the Care and Use of Laboratory Animals published by the US National Institutes of Health (NIH Publication no. 85-23, revised 1996).

### Diabetic animal models

Male Sprague Dawley rats, 6–8 weeks of age, were kept in the Animal Center of Nanfang Hospital according to the policy of the Committee for Animal Usage. Rat models of diabetes were induced with a single intraperitoneal injection of either streptozotocin (65 mg/kg; S0130; Sigma-Aldrich) in 0.1 M citrate buffer (pH 4.5) or 0.1 M citrate buffer according to the protocol described previously.^34, 50, 51^ Rats with a blood glucose level over 16.7 mmol/l were considered diabetic and thus included in the study, followed by treatment with the intraperitoneal administration of miR-27ai or miR-27am (4 ng/mm^3^) or the appropriate scrambled control RNAs every day till killing at week 12 of diabetes.

### Groups and sample collection

Six groups of rats were used: (1) normal control (NC, *n*=6), (2) diabetic control (DM, *n*=7), (3) diabetic rats treated with NC miRNA inhibitor (DM_miR-iNC, *n*=8), (4) diabetic rats treated with miR-27ai (DM_miR-27ai, *n*=7), (5) diabetic rats treated with NC miRNA mimic (DM_miR-NC, *n*=6) and (6) diabetic rats treated with miR-27a mimic (DM_miR-27am, *n*=6). No adverse or toxic effects were observed.

Blood glucose level was measured every week. The treatment continued until the rats were killed. At 12 weeks after the induction of diabetes, rats were housed in metabolic cages and urine was collected for the determination of UAER and UACR. Then, rats were anesthetized with pentobarbital sodium (P3761, 30 mg/kg, Sigma-Aldrich) and the venous blood was drawn from the eye orbit of rats before killing. Left kidneys were fixed by retrograde aortic perfusion using phosphate-buffered saline (PBS, pH 7.4). Cortex of the left kidneys was obtained for electron microscopy analysis and the rest was fixed in 10% formalin in PBS for 24 h, rinsed in buffer, dehydrated and embedded in paraffin for histological analysis. The non-perfused right kidney was cut into small pieces, snap-frozen and stored at −80 °C for further analysis. Rats were killed by exsanguination without any previous intervention following guidelines recommended from the Animal Research: Reporting *In Vivo* Experiments (ARRIVE) (http://www.nc3rs.org.uk/page.asp?id=1357). All efforts were made to minimize suffering.

Blood glucose, BUN, serum and urine creatinine were analyzed using a Beckman Coulter AU480 Chemistry Analyzer (Beckman, Brea, CA, USA). Urine albumin was determined with an enzyme-linked immunosorbent assay kit specific for rat albumin (E111-125, Bethyl Laboratories, Montgomery, TX, USA). Ccr was calculated as urinary creatinine (umol/l) × urine volume (ml/min)/Scr (umol/l), and was expressed as ml/min/kg. All experiments were repeated in triplicate.

### Transmission electron microscopy

Several 1-mm cubes from cortex of left kidneys were cut, placed in 2.5% glutaraldehyde for at least 4 h washed with cacodylate buffer, postfixed in 1% osmium tetroxide and block-stained in uranyl acetate before embedding in Poly/Bed812 resin (Polysciences, Inc., Warrington, PA, USA). Ultrathin sections were obtained from at least three randomly selected glomeruli from each animal, stained with uranyl acetate and lead citrate and examined using a Hitachi 7,700 transmission electron microscope (Tokyo, Japan). Representative micrographs of the podocytes from each rat were examined and imaged at a magnification of × 4000.

### Laser capture microdissection (LCM)

Fresh snap-frozen rat kidney tissues were embedded in optimal cutting temperature (OCT) compound (Sakura Finetek Japan Co., Ltd, Tokyo, Japan). Ten-*μ*m thick cryosections were cut and mounted on Membrane slides (Muster Membrane Slides 1.0 polyethylene naphthalate (PEN)-covered glass slide; 000757-11, Zeiss, Jena, Germany). On each unstained membrane slide, 50–100 individual glomeruli were laser microdissected and collected using the PALM MicroBeam LCM system (Zeiss) for RNA and protein extraction as described previously.^50^

### Quantitative real-time RT-PCR analysis

Total RNA from podocytes and microdissected glomeruli were extracted using TRIzol reagent (MRC, Cincinnati, OH, USA). First-strand cDNA was synthesized using 2 *μ*g of total RNA treated with Moloney murine leukemia virus reverse transcriptase (Promega) according to the manufacturer's instructions. qRT-PCR analysis was performed in triplicate with Power PCR SYBR Green Master Mix (Applied Biosystems, Carlsbad, CA, USA) using the ABI PRISM 7500 FAST Real-TIME PCR System (Applied Biosystems) with results normalized to *β*-actin expression. The ΔΔCT method was used to calculate relative expression. Primer sequences used in qRT-PCR are shown in Supplementary Tables 1 and 2.

To assess the level of miRNA expression, total RNA extracted from the cells, human plasma samples, rat glomerular tissues or plasma samples was reversely transcribed into cDNA using miRScript PCR System and then analyzed by qRT-PCR with the miScript SYBR Green PCR Kit using the specific miR-27a miScript Primer Assays (Qiagen) according to the manufacturers' instructions. Expression levels were normalized to the average of U6-snuRNA. MiR-27a levels were calculated as fold change (2^−^^ΔΔCT^) with respect to normal controls. The mean value of miR-27a expression in glucose-free cultured cells was used as the calibrator. Target-specific reverse transcription and Taqman miRNA assays were performed using the Hairpin-it miRNA qPCR Quantitation Kit (GenePharma, Suzhou, China) according to the protocol. The reactions were performed using the ABI PRISM 7500 FAST Real-TIME PCR System (Applied Biosystems) with results normalized to U6-snuRNA expression. The 2^−ΔΔCt^ method was used to calculate the relative expression. All experiments were performed in triplicate.

### Western blot analysis

Lysates from podocytes and microdissected glomeruli from each experimental group were separated in parallel on two 10% denaturing sodium dodecyl sulfate-polyacrylamide gels, transferred onto nitrocellulose membranes, blocked with 5% nonfat milk in 0.1% tris-buffered saline with Tween-20 (TBST) and probed using primary antibodies against PPAR*γ* (phospho S112) (1:100, Santa Cruz Biotechnology, Santa Cruz, CA, USA), PPAR*γ* (1:100, ab45036, Abcam, Cambridge, UK), non-phospho (active) *β*-catenin (Ser45) (D2U8Y, 1:50, #19807, Cell Signaling Technology, Beverly, MA, USA), active *β*-catenin (05-665; EMD Millipore, Billerica, MA, USA), snail1 (1:100, ab53519, Abcam), *α*-SMA (1:100, ab5694, Abcam), NPHS2 (1:100, ab50339, Abcam), synaptopodin (1:50, 39067, GeneTex, San Antonio, TX, USA) and *β*-actin (1:200, ab6276, Abcam) at 4 °C overnight. After extensive washing in TBST buffer, the secondary antibody (horseradish peroxidase-labeled IgG anti-rabbit/mouse antibody, Invitrogen, Cambridge, MA, USA) was used at 1:3000 dilution for 1 h at room temperature. The supersignal-enhanced chemoluminescent substrate (Pierce Biotechnology, Inc., Rockford, IL, USA) was applied to the probed membrane and exposed for 10 min before the protein bands were visualized on radiograph films (Super Rx, Fuji Photo Film, Tokyo). Quantification was performed by measurement of the intensity of the bands using ImageJ analysis software (National Institute of Health, Bethesda, MD, USA).

### Patients and renal biopsy studies

A total of 62 formalin-fixed paraffin-embedded renal biopsy samples were obtained from type 2 diabetic patients including 46 from the Department of Renal Pathology in King Medical Diagnostics Center in Guangzhou from 2012 to 2014 and 16 from the Division of Nephrology in the First Affiliated Hospital of Inner Mongolia Medical University in Hohhot from 2010 to 2015. The inclusion criteria were: (1) type 2 diabetic patients with no history of using renal toxic or herbal medicine; (2) the indications for performing the renal biopsy were proteinuria with or without microscopic hematuria and fast drop in renal function; (3) type 2 diabetic patients with no complications of other renal diseases. The Ethics Committee from King Medical Diagnostics Center and Inner Mongolia Medical University specifically approved the use of patient tissue samples in this study and written informed consent was obtained from each patient.

In all specimens, the morphological diagnosis of DN was confirmed by two individual renal pathologists (JG and XB). Normal kidney tissues (*n*=13) were obtained from healthy transplant donor kidney biopsies and thus served as controls. Serum samples from 30 DN patients and 30 healthy volunteers were collected.

### miR-27a ISH

ISH for miR-27a (50 nM) was performed as described previously.^52^ Frozen kidney samples from rats were fixed in 4% paraformaldehyde in 0.1 M PBS (pH 7.4) containing 1/1000 diethylprocarbonate overnight at 4 °C before staining. Samples were then placed in 1 mol/l sucrose solution overnight at 4 °C and embedded in OCT compound at −80 °C. Ten-*μ*m-thick cryosections were prepared and the ISH analysis was performed using digoxygenin (DIG)-labeled miRCURY Locked Nucleic Acid (LNA) microRNA Detection Probes (Exiqon, Vedbaek, Denmark) according to the manufacturer's protocol. Briefly, slides were pre-digested with proteinase-K (15 *μ*g/ml) at 37 °C for 8 min, pre-hybridized at 57 °C for 15 min, hybridized with double-carboxyfluorescein (FAM) labeled LNA probes (Exiqon A/S) diluted in hybridization buffer (PerfectHyTM Plus Hybridization Buffer; Sigma-Aldrich) at 56 °C in a humidified chamber for 2 h in a humidified chamber. After stringent washes with saline-sodium-citrate buffer (pH 7.0), the probes were detected with alkaline phosphatase-conjugated sheep anti-FAM Fab fragments, followed by incubation in 4-nitroblue tetrazolium and 5-bromo-4-chloro-3′-indolylphosphate (NBT/BCIP) solution (Roche, Basel, Switzerland) for 60 min. The signal resulted in a dark blue staining. Photographs were taken under a BX-51 light microscope (Olympus, Tokyo, Japan) fitted with a DFC550 color video camera (Leica Microsystems, Wetzlar, Germany) using appropriate filters.

### Immunofluorescence and immunohistochemical analysis

Podocytes, tissue samples from rats and patients were labeled with antibodies to pPPAR*γ* (1:100), active beta-catenin (1:100), snail1 (1:100), *α*-SMA (1:100), synaptopodin (1:1000) and WT1 (1:100). For immunofluorescence staining, Alexa Fluor 594-conjugated goat anti-mouse IgG and Alexa Fluor 488-conjugated goat anti-rabbit IgG (1:1000, Invitrogen, Cambridge, MA, USA) were used for secondary antibodies, nuclei were counterstained with 4′,6-diamidino-2-phenylindole (DAPI, Sigma-Aldrich) and coverslipped with aqueous mounting medium (CTS011, BD Bioscience, Minneapolis, MN, USA). For immunohistochemistry, EnVision Detection Systems Peroxidase/diaminobenzidine (DAB), rabbit/mouse kit (K4065, Dako, Carpinteria, CA, USA) was used. Nuclei were counterstained with hematoxylin and coverslipped with Permount mounting medium (00-4960-56, eBioscience, San Diego, CA, USA).

Samples were evaluated semiquantitatively by systematically selecting without bias 20 fields for analysis. Immunofluorescence images were taken with a FV1000-IX81 confocal laser scanning microscope (Olympus) with appropriate filters. Staining intensity was measured using ImageJ analysis software (ImageJ 1.44, National Institute of Health). The number of cells positive for WT-1 was counted in all glomeruli at × 400 magnification in each section and the mean number was recorded as podocyte number in each sample. Immunohistochemical images were taken under a BX-51 light microscope (Olympus) fitted with a DFC550 color video camera (Leica Microsystems) using appropriate filters. PBS instead of primary antibodies served as a NC.

### Statistical analyses

Data are presented as mean±S.D. Statistical significance between groups was evaluated using independent samples *T*-test or one-way ANOVA followed by post-hoc least-significant difference test. All statistical tests were performed using SPSS 12.0 (SPSS, Inc., Chicago, IL, USA). The significance level is set at 0.05 to indicate statistical significance.

## Figures and Tables

**Table 1:** Parameters for diabetic rats treated with miR-27a inhibitor at week 12

**Variables**	**DM_miR-iNC (*****n*****=8)**	**DM_miR-27ai (*****n*****=7)**
Scr (*μ*mol/l)	124.26±6.38	70.13±2.17*
Serum BUN (mmol/l)	17.58±4.56	10.25±1.31*
Blood glucose (mmol/l)	24.58±1.23	25.68±1.75
UAER (*μ*g/min)	1.69±0.63	0.88±0.08^#^
UACR (*μ*g/mmol)	25.78±2.45	13.95±1.53^#^
Ccr (ml/min/kg)	4.14±0.45	8.26±0.75^#^

Abbreviations: BUN, blood urea nitrogen; Ccr, creatinine clearance rate; DM_miR-iNC, diabetic rats treated with miRNA inhibitor negative control; DM_miR-27ai, diabetic rats treated with miR-27a inhibitor; Scr, serum creatinine; UACR, urine albumin to creatinine ratio; UAER, urine albumin excretion rate

*P<0.01; ^#^*P*<0.001

**Table 2:** Parameters for diabetic rats treated with miR-27a mimics at week 12

**Variables**	**DM_miR-NC (*****n*****=6)**	**DM_miR-27am (*****n*****=6)**
Scr (*μ*mol/l)	118.35±5.19	130.27±6.58*
Serum BUN (mmol/l)	16.79±3.37	20.58±3.13*
Blood glucose (mmol/l)	25.45±0.37	26.77±1.34
UAER (*μ*g/min)	1.75±0.31	3.02±0.61^#^
UACR (*μ*g/mmol)	26.15±2.25	60.17±3.56^#^
Ccr (ml/min/kg)	3.55±0.42	0.72±0.06^#^

Abbreviations: BUN, blood urea nitrogen; Ccr, creatinine clearance rate; DM_miR-NC, diabetic rats treated with miRNA mimics negative control; DM_miR-27am, diabetic rats treated with miR-27a mimics; Scr, serum creatinine; UACR, urine albumin to creatinine ratio; UAER, urine albumin excretion rate

*P<0.01; ^#^*P*<0.001
