# Correction: MicroRNA-27a promotes podocyte injury via PPARγ-mediated β-catenin activation in diabetic nephropathy

**DOI:** 10.1038/s41419-018-0637-3

**Published:** 2018-05-29

**Authors:** Zhanmei Zhou, Jiao Wan, Xiaoyan Hou, Jian Geng, Xiao Li, Xiaoyan Bai

**Affiliations:** 1 Division of Nephrology, Nanfang Hospital, Southern Medical University, National Clinical Research Center for Kidney Disease, State Key Laboratory of Organ Failure Research, Guangdong Provincial Institute of Nephrology, Guangzhou, Guangdong China; 2 Department of Nephrology, The First Affiliated Hospital, Inner Mongolia Medical University, Hohhot, Inner Mongolia China; 3 Department of Pathology, Nanfang Hospital, Southern Medical University, Guangzhou, Guangdong China; 4 Department of Emergency, Nanfang Hospital, Southern Medical University, Guangzhou, Guangdong China

**Correction to:** *Cell Death and Disease* **8**, e2658 (2017); 10.1038/cddis.2017.74; published online 9 March 2017

Since the publication of this article, the authors identified an omission in the funding information. The original funding information was as follows:

This study was supported by National Nature and Science Young Investigator Grant (no. 81100496) from the National Natural Science Foundation of China, Special Fund from Chinese Society of Nephrology (no. 13030370422), Guangdong Natural Science Foundation (no. 2016A030313581) and Distinguished Young Scholar Fund from Nanfang Hospital (no. 2015J009) to X.B. We thank Guangzhou King Medical Diagnostics Center for providing human renal biopsy samples. We gratefully acknowledge all lab members for their technical assistance.

The authors would also like to acknowledge the following:

Guangzhou Science and Technology Planning Project-Key Projects of Scientific Research (201607020019) and Distinguished Young Scholar Fund from Nanfang Hospital (no. 2015J009) to X.B.

The authors apologise for any inconvenience caused.

