# Supplementary material for: MicroRNA-27a promotes podocyte injury via PPARγ-mediated β-catenin activation in diabetic nephropathy
Source: Cell Death Dis. 2017 Mar 9;8(3):e2658–. doi: 10.1038/cddis.2017.74 (PMC5386567; doi:10.1038/cddis.2017.74)
Supplement: Supplementary Information [file cddis201774x1.docx]

**Supplementary Information**

**MicroRNA-27a promotes podocyte injury via PPARγ-mediated β-catenin activation in diabetic nephropathy**

**Zhanmei Zhou^1, ‡^, Jiao Wan^1, ‡^, Xiaoyan Hou^1, 2, ‡^, Jian Geng^3^, Xiao Li^4^, Xiaoyan Bai^1, *^**

^1^Division of Nephrology, Nanfang Hospital, Southern Medical University, National Clinical Research Center for Kidney Disease, State Key Laboratory of Organ Failure Research, Guangdong Provincial Institute of Nephrology, Guangzhou, Guangdong, PR China; ^2^Department of Nephrology, The First Affiliated Hospital, Inner Mongolia Medical University, Hohhot, Inner Mongolia, PR China; ^3^Department of Pathology, Nanfang Hospital, Southern Medical University, Guangzhou, Guangdong, PR China; ^4^Department of Emergency, Nanfang Hospital, Southern Medical University, Guangzhou, Guangdong, PR China.

**List of Contents:**

**Supplementary Tables**

**Supplementary Figures**

**Supplementary Table 1** Primer sets used in real time RT-PCR

| **Gene (mouse)** |  |  |
| --- | --- | --- |
| ***Pparγ*** | Forward Primer  Reverse Primer | 5’-TGCCTTCCCTGTGAACTGAC-3’  5’-TGGGGAGAGAGGACAGATGG-3’ |
| ***Ctnnb1***  ***(β-catenin)*** | Forward Primer  Reverse Primer | 5’-GTCAGTGCAGGAGGCCG-3’  5’-GGCCATGTCCAACTCCATCA-3’ |
| ***Snail1*** | Forward Primer  Reverse Primer | 5’-AGTTGACTACCGACCTTGCG-3’  5’-TGCAGCTCGCTATAGTTGGG-3’ |
| ***Acta2 (α-SMA)*** | Forward Primer  Reverse Primer | 5’-TCCTGTTTCGGGAGCAGAAC-3’  5’-AGCTGGCCGTTCACTCTAAC-3’ |
| ***Actb (β-actin)*** | Forward Primer  Reverse Primer | 5'-CCACCATGTACCCAGGCATT-3'  5'-AGGGTGTAAAACGCAGCTCA-3' |

**Supplementary Table 2** Primer sets used in real time RT-PCR

| **Gene (rat)** |  |  |
| --- | --- | --- |
| ***Pparγ*** | Forward Primer  Reverse Primer | 5’-CCTCGAGGACACCGGAGA-3’  5’-CACGGAGCTGATCCCAAAGT-3’ |
| ***Ctnnb1***  ***(β-catenin)*** | Forward Primer  Reverse Primer | 5’-GACGGAGGAAGGTCTGAGGA-3’  5’-TGGCCATGTCCAACTCCATC-3’ |
| ***Snai1 (Snail1)*** | Forward Primer  Reverse Primer | 5’-GAGCCCAGGCAGCTATTTCA-3’  5’-TGGGAGACACATCGGTCAGA-3’ |
| ***Acta2 (α-SMA)*** | Forward Primer  Reverse Primer | 5’-AAAGCAAGTCCTCCAGCGTT-3’  5’-TAGTCCCGGGGATAGGCAAA-3’ |
| ***Actb (β-actin)*** | Forward Primer  Reverse Primer | 5'-GTCATTCCAAATATGAGATGCGT-3'  5'-GCTATCACCTCCCCTGTGTG-3' |

**Supplementary Figure 1**

**
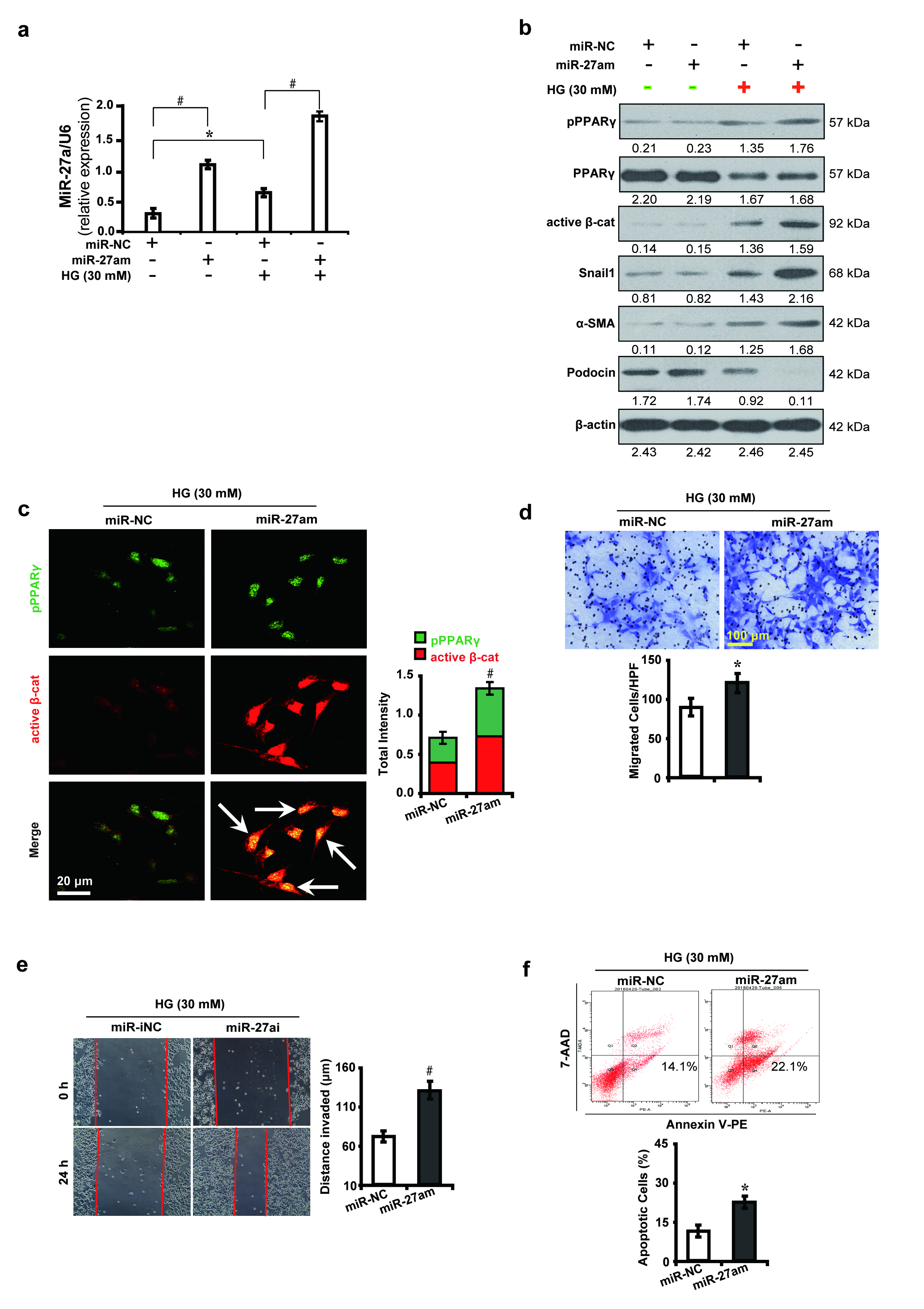
**

**Supplementary Figure 1** MiR-27a mimics promotes podocyte injury via PPARγ-mediated β-catenin activation in high glucose. (**a**) qRT-PCR analysis shows miR-27am augmented miR-27a expression in HG cultured podocytes. (**b**) Representative Western blotting shows the expression of phosphorylated and total PPARγ and β-catenin target genes in various conditions as indicated. (**c**) Immunofluorescence staining shows the upregulation of phosphorylated PPARγ (green) and active β-catenin (red) by miR-27am. Arrows indicate colocalization (yellow). Scale bar, 20 μm. (**d**) Transwell migration assay and quantitative data show increased migration of HG cultured podocytes. Scale bar, 100 μm. (**e**) Wound healing assay and quantitative data show increased invasion of HG cultured podocytes. (**f**) Flow cytometric analysis shows increased podocyte apoptosis.

**Supplementary Figure 2**


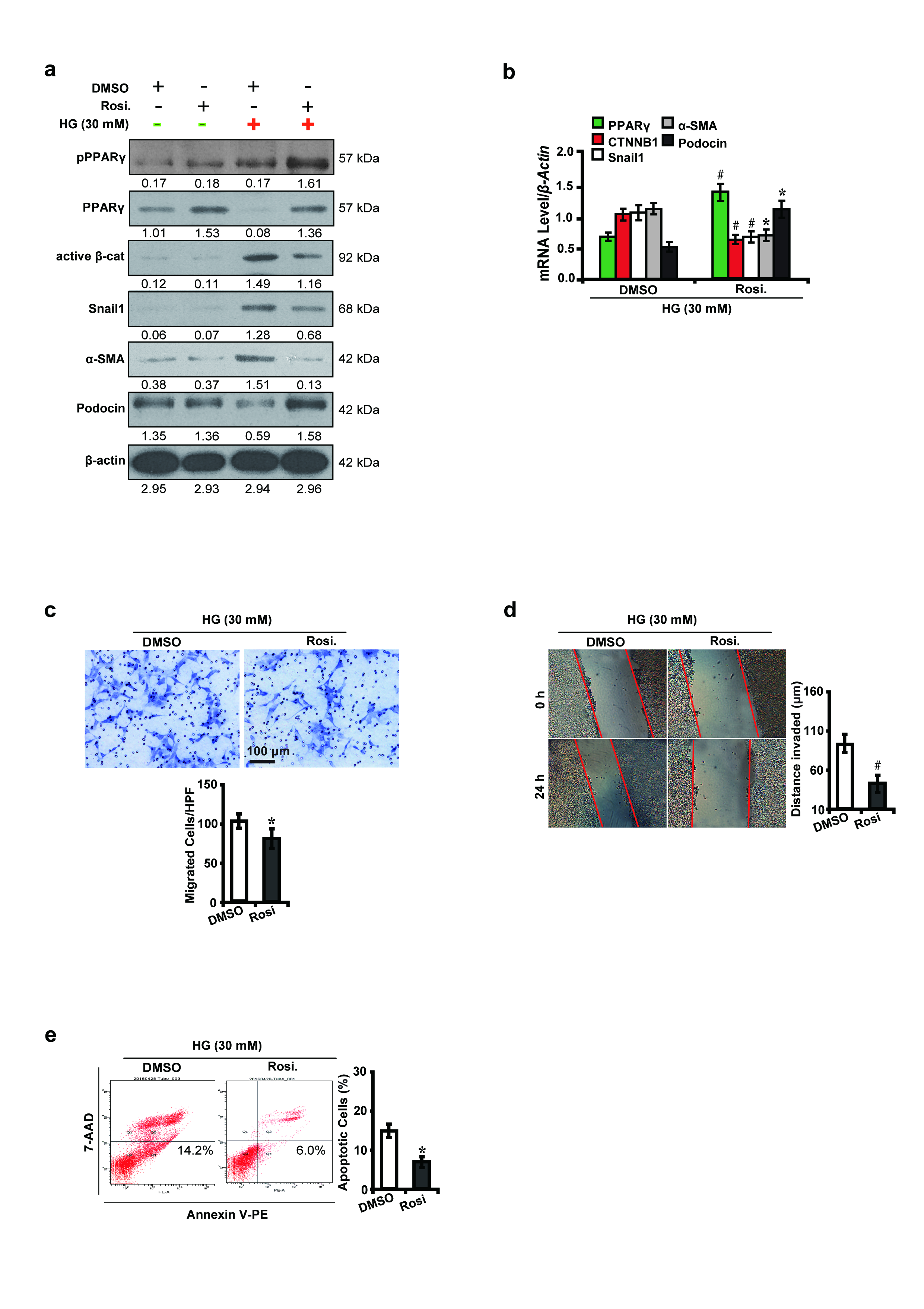


**Supplementary Figure 2** PPARγ overexpression diminishes β-catenin activation and ameliorates podocyte injury in high glucose. (**a**) Western blotting shows the expression of PPARγ and β-catenin target genes upon rosiglitazone treatment as indicated. (**b**) qRT-PCR analysis shows Rosi. increased PPARγ and Podocin but decreased β-catenin target genes. (**c**) Transwell migration assay shows decreased migration. Scale bar, 100 μm. (**d**) Wound healing assay shows decreased invasion. (**e**) Summarized data shows decreased podocyte apoptosis by flow cytometric analysis. **P*<0.05; #*P*<0.001.
